# The influence of stimulus repetition on duration judgments with simple stimuli

**DOI:** 10.3389/fpsyg.2015.01213

**Published:** 2015-08-18

**Authors:** Teresa Birngruber, Hannes Schröter, Rolf Ulrich

**Affiliations:** Department of Psychology, University of TübingenTübingen, Germany

**Keywords:** time perception, repetition, novelty, nonwords, duration judgment

## Abstract

Two experiments investigated the effects of stimulus repetition vs. stimulus novelty on perceived duration. In a reminder task, a standard and a comparison stimulus were presented consecutively in each trial, and the comparison was either a repetition of the standard or a different stimulus. Pseudowords (Experiment 1) or strings of consonants (Experiment 2) were used as stimuli and the inter-stimulus interval (ISI) between the standard and the comparison was either constant or variable. Participants were asked to judge whether the comparison was shorter or longer than the standard. In both experiments, we observed shorter judged durations for repeated than for novel comparisons whereas the manipulation of the ISI had no pronounced effects on duration judgments. The finding of shorter duration judgments for repeated as compared to novel nonwords replicates the results of a previous study (Matthews, 2011) which employed highly complex stimulus material. The present study shows that changes of simple, semantically meaningless stimuli are sufficient to result in a shorter perceived duration of repeated as compared to novel stimuli.

## 1. Introduction

Human time perception is known to be influenced by many non-temporal aspects (Eagleman, 2008; Grondin, 2010). For example, perceived duration not only depends on physical time but also on the sensory modality stimuli are presented in Goldstone and Lhamon (1974), Wearden et al. (1998), low-level stimulus features (such as contrast: Matthews et al., 2011, or stimulus size: Thomas and Cantor, 1976; Rammsayer and Verner, 2015), and the emotional context of stimulus presentation (Droit-Volet et al., 2011).

Another context effect is the so-called temporal oddball effect. It describes the phenomenon that the duration of deviant stimuli (oddballs) within a stream of homogenous standards is commonly judged as being longer than the duration of the standards (Tse et al., 2004; Pariyadath and Eagleman, 2007; Chen and Yeh, 2009; New and Scholl, 2009; Schindel et al., 2011; Kim and McAuley, 2013; Birngruber et al., 2014). This result from the “stream-based” oddball paradigm has mostly been interpreted as a temporal overestimation of oddballs. But since only relative judgments between standards and oddballs are required, it could just as well reflect a temporal underestimation of standards (see Birngruber et al., 2015, for a study including judgments of standards as well as oddballs).

Matthews (2011) has shown that even a single repetition of a stimulus can result in a shortened judged duration of this stimulus as compared to a novel stimulus. In his experiments, only two stimuli, first a standard and then a comparison, were presented in each trial and had to be compared in duration (reminder task, see also Ulrich et al., 2006). Naturalistic photographs of different content, e.g., social scenes, nature, objects, and buildings were used as stimuli. The comparison could either be a repetition of the standard or a novel photograph (never encountered before). Matthews observed that repeated comparisons were systematically underestimated compared to novel ones.

The results by Matthews (2011) provide evidence that a single stimulus repetition influences duration judgments. The stimulus material used in this study was rather complex and thus differed on many levels of information: content, categories, color, texture, contrast, etc. Consequently, all these features remained the same between standards and repeated comparisons whereas there were multi-level differences between standards and novel comparisons. In order to examine whether this effect persists even without high-level information, we designed a conceptual replication of Matthews' study using nonwords as stimuli. Nonwords are much simpler than photographs and the only difference for repeated as compared to novel comparisons is whether the letter string of the nonword is repeated or changed. Whether the letter string itself represents rather a low- or a high-level feature is not easy to decide. On the one hand, individual letters obviously vary in shape and nonwords might therefore differ slightly in spatial frequency and overall luminance. On the other hand, many low-level features of nonwords can be easily controlled (e.g., size, color, contrast) and nonwords have per definition no semantic meaning. We chose nonwords as stimuli because high-level information and low-level differences could be minimized while a straight-forward manipulation of repetition was possible. If repetition as compared to a change of information is sufficient to influence perceived duration even if semantic meaning is absent and low-level differences are minimized, repeated nonwords should be judged as being shorter than novel ones.

It should be noted, however, that Matthews recently replicated the repetition effect for a more abstract set of stimuli himself (Matthews, 2015). In Experiments 5 and 6 of this study, nine icons of abstract two-color patterns were combined to 3 × 3 grids and presented as standards and comparisons in a reminder task. While these stimuli had no semantic meaning either, they still contained color, luminance, and shape changes and therefore might have differed on multiple levels. In contrast, the present study examined whether the repetition effect would even generalize to nonword stimuli which are composed of over-learned elements (i.e., letters) and differ only minimally in low-level features.

Furthermore, to address a different issue, we manipulated whether the inter-stimulus intervals between the presentation of the standard and the comparison were predictable or not. Tse et al. (2004) have argued that a fixed temporal structure within a trial might induce rhythm which could interact with duration perception. To investigate this possibility, we presented constant inter-stimulus intervals (as in Matthews, 2011, 2015) in one half of the experiment and variable inter-stimulus intervals (as in Tse et al., 2004) in the other half. If a strictly predictable temporal structure would facilitate rhythmic processing, temporal discrimination sensitivity should be better with constant than with variable inter-stimulus intervals.

## 2. Experiment 1

### 2.1. Method

#### 2.1.1. Participants

The data of 32 volunteers (22 female, 29 right-handed), aged between 21 and 51 years (*M* = 24.2 years) entered the analyses. All participants had normal or corrected-to-normal vision, and all received course credit. The experimental session lasted approximately 40 min. Eight additional participants took part in the experiment but had to be excluded due to DL (difference limen) measures larger than 200 ms in at least one of the four conditions. Since the corresponding psychometric functions were almost flat, the PSE estimates were rather unreliable and we therefore considered these data sets to be uninformative with respect to the research question. All participants gave informed consent.

#### 2.1.2. Apparatus and stimuli

The experiment was programmed in MATLAB® using the Psychophysics Toolbox extension (Brainard, 1997; Pelli, 1997) and presented via a PC with standard VGA monitor (1024 × 768 pixels, 150 Hz). As nonword stimuli, 104 pseudowords (pronounceable but meaningless letter strings) were taken from the *Verbaler Lerntest* (verbal learning test, Sturm and Willmes, 1999). These pseudowords were the low-associative subset of the items used in this memory test (see Sturm and Willmes, 1999). This means that they were rated as being unlikely to be associated with actual German words. The pseudowords were comprised of six letters and two syllables, e.g., “MEILEG,” “DRISIT,” or “GELPOS.” All pseudowords were presented in capital letters in white font color on a black background, were about 1.8 cm long (2.6° of visual angle), and were always presented in the center of the screen. For each participant, 81 items were randomly selected from the pool of 104 pseudowords. The “X” and “M” keys of a standard German keyboard served as response keys.

#### 2.1.3. Procedure

**Figure 1:**
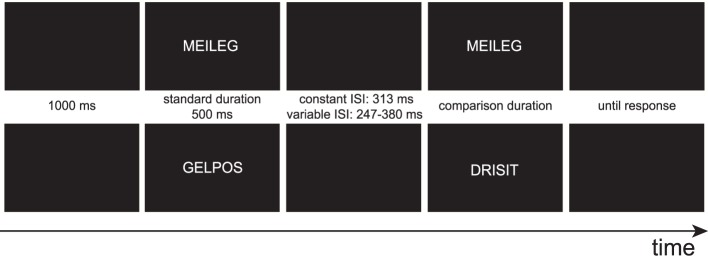
**Trial structure of Experiment 1**. An example of a repeated (novel) trial can be seen at the top (bottom). The inter-stimulus interval was either fixed in the constant ISI condition or varied in the variable ISI condition. Comparison durations varied across nine durations between 313 and 687 ms and the participants' task was to judge whether the comparison was shorter or longer than the standard.

The experiment was run in a sound-attenuated, dimly illuminated room. An illustration of the trial structure can be found in Figure 1. Each trial started with a blank black screen which was presented for 1000 ms. Then, two stimuli were presented one at a time. The first stimulus (standard) was always presented for 500 ms, the second stimulus (comparison) was presented for one of nine comparison durations: 313, 360, 407, 453, 500, 547, 593, 640, or 687 ms. The two stimuli were separated by an inter-stimulus interval (ISI). In the constant ISI condition, the ISI was 313 ms; in the variable ISI condition, ISIs were randomly selected from the following five durations: 247, 280, 313, 346, and 380 ms. In half of the trials, the comparison was identical to the standard (repeated condition), whereas in the other half of the trials, a different pseudoword was shown (novel condition). The participants were instructed to make a judgment about whether the comparison was shorter or longer than the standard, irrespective of condition. After the participant's key press, the next trial started.

The experiment was segmented in one practice block and 12 experimental blocks. The practice block was comprised of 12 trials (six repeated and six novel trials). The items for the practice block were chosen randomly from the pool of pseudowords. Six of the 12 experimental blocks realized the constant ISI condition while the other six blocks realized the variable ISI condition. The ISI condition was blocked and the practice block was of the same ISI condition as the first half of the experiment. Each experimental block was comprised of 54 trials (27 repeated and 27 novel trials), thus each block included all 81 items that were pre-selected for each participant. The same 81 items were presented in each block, but whether individual items appeared in a repeated or novel trial was randomized. The fact that items were therefore presented several times throughout the experiment should not be problematic as repetition effects on time perception seem to be quite short-lived (see Matthews, 2011, Experiment 2 and Matthews, 2015, Experiments 5 and 6). Short breaks were included between experimental blocks and once within each block (every 27 trials). In total, 648 experimental trials and 12 practice trials were processed.

#### 2.1.4. Design and data analysis

The experiment had a 2 × 2 factorial design, resulting from the orthogonal combination of the within-subject factors repetition (repeated vs. novel) and ISI (constant vs. variable). The order of the ISI blocks (constant first vs. variable first) and the judgment-to-key assignment (left-shorter, right-longer vs. left-longer, right-shorter) were counterbalanced across participants.

Logistic functions were fitted to the data of each condition and for each participant. The point of subjective equality (PSE) was computed from each function as a measure of perceived duration. The PSE indicates the comparison duration which appears to be just as long as the standard. Larger PSEs indicate that the participant tends to perceive the comparison as shorter than the standard. In addition, DL was also computed from these functions as a measure of discrimination sensitivity. Larger DLs indicate poorer temporal discrimination.

Finally, we analyzed response times (RTs) (see Birngruber et al., 2015, for another application of RT analyses in duration judgment tasks). First, we excluded all trials with RTs which were larger than 4000 ms because we considered them outliers (this led to the exclusion of 105 trials which is < 0.6% of all trials). Then we computed mean RT as a function of comparison duration. Typically, RT in choice paradigms like the present shorter-longer-judgment task increases with discrimination difficulty (Birren and Botwinick, 1955; Sternberg, 1969). Thus, mean RT as a function of comparison duration should result in an inverted U-shaped function showing that participants need more time to decide whether the comparison was shorter or longer than the standard if the comparison duration is close to the PSE. To quantify the location of this inverted U-shaped function, we determined its first moment using the waveform moment analysis (Cacioppo and Dorfman, 1987; Ulrich et al., 1995). This location parameter represents the respective comparison duration at which the mean of the function is located^1^. This measure assesses the comparison duration that is most difficult to discriminate. We will, therefore, refer to this parameter as the point of maximal uncertainty (PMU). We determined PMU separately for each participant and each condition. A significance level of 0.05 was set for all significance tests and *p*-values were Greenhouse–Geisser corrected where appropriate.

### 2.2. Results and discussion

**Figure 2:**
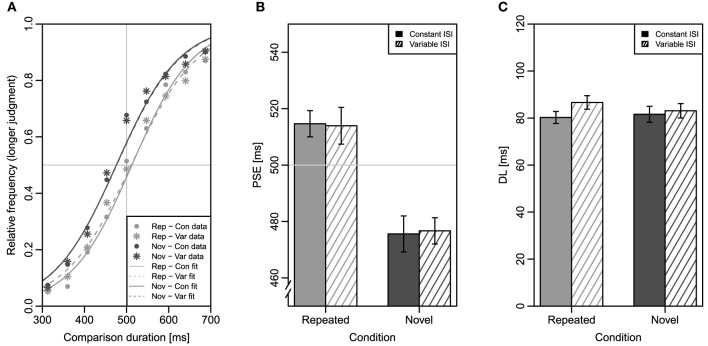
**Results of Experiment 1**. Error bars represent ±1 standard error for within-subjects designs according to Morey (2008). **(A)** Mean relative frequencies of longer judgments as a function of comparison duration, in the four conditions (symbols) and fitted logistic functions for all participants' data (lines). Note that this plot is only for illustration; individually fitted logistic functions for each participant were the basis of the statistical analyses. The horizontal light gray line indicates the 50% point; the vertical light gray line indicates the standard duration of 500 ms. **(B)** Mean point of subjective equality (PSE) in the four conditions. **(C)** Mean difference limen (DL) in the four conditions. Rep, repeated condition; Nov, novel condition; Con, constant inter-stimulus interval; Var, variable inter-stimulus interval.

Figure 2A shows mean relative frequencies of “longer”-judgments as a function of comparison duration together with the fitted logistic function for each of the four conditions. Note that these functions are only for illustration, while the individual PSE and DL-values that entered the following analyses were derived from individually fitted psychometric functions.

A two-factor repeated measures ANOVA with the factors repetition (repeated vs. novel) and ISI (constant vs. variable) was conducted on PSE. Figure 2B depicts mean PSE as a function of the two factors. A significant main effect of repetition was present, *F*_(1, 31)_ = 23.60, *MSE* = 1975, *p* < 0.001, $\eta_{p}^{2}=0.43$, indicating that mean PSE for repeated comparisons (514 ms) was larger than mean PSE for novel comparisons (476 ms). The main effect of ISI was not significant, *F* < 1, because PSE was identical for constant and variable ISIs (495 ms). The ANOVA revealed no interaction of the two factors, *F* < 1^2^.

A two-factor repeated measures ANOVA with the same two factors was performed on DL. Figure 2C depicts mean DL. Neither the main effect of repetition, *F* < 1, nor the main effect of ISI, *F*_(1, 31)_ = 1.27, *MSE* = 391.0, *p* = 0.269, $\eta_{p}^{2}=0.04$, were significant, indicating that discrimination sensitivity was almost identical for repeated and novel comparisons (83 and 82 ms) and for constant and variable ISIs (81 and 85 ms). The interaction of the factors was not significant either, *F*_(1, 31)_ = 1.12, *MSE* = 171.5, *p* = 0.298, $\eta_{p}^{2}=0.03$.

**Figure 3:**
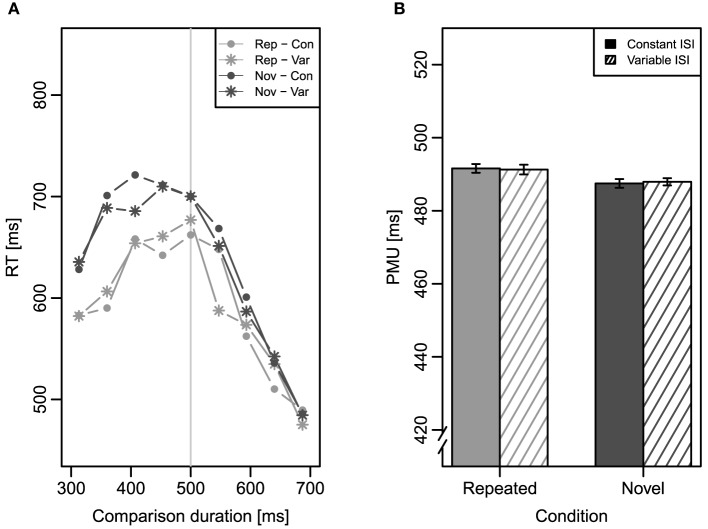
**Results of Experiment 1**. Error bars represent ±1 standard error for within-subjects designs according to Morey (2008). **(A)** Mean response time (RT) as a function of comparison duration in the four conditions. The vertical light gray line indicates the standard duration of 500 ms. **(B)** Mean point of maximal uncertainty (PMU) in the four conditions. Rep, repeated condition; Nov, novel condition; Con, constant inter-stimulus interval; Var, variable inter-stimulus interval.

To analyze RT, we conducted a three-factor repeated measures ANOVA with the factors repetition (repeated vs. novel), ISI (constant vs. variable), and comparison duration (nine levels, 313–687 ms) on mean RT. Mean RT as a function of comparison duration is depicted in Figure 3A. The main effect of repetition was significant, *F*_(1, 31)_ = 15.26, *MSE* = 23, 678, *p* < 0.001, $\eta_{p}^{2}=0.33$, showing that RTs were generally longer for novel comparisons (637 ms) than for repeated ones (595 ms). As expected, RTs also changed across comparison durations causing a significant main effect of comparison duration, *F*_(8, 248)_ = 26.96, *MSE* = 24, 112, *p* < 0.001, $\eta_{p}^{2}=0.47$, and a significant interaction of repetition and comparison duration, *F*_(8, 248)_ = 2.26, *MSE* = 10, 772, *p* = 0.044, $\eta_{p}^{2}=0.07$. No other effects of this ANOVA reached significance.

The PMUs which were calculated individually for each participant, served as the dependent variable of a two-factor repeated measures ANOVA with the factors repetition and ISI. Mean PMU for the four conditions can be found in Figure 3B. The significant main effect of repetition, *F*_(1, 31)_ = 9.12, *MSE* = 48.80, *p* = 0.005, $\eta_{p}^{2}=0.23$, confirmed that the comparison duration-RT functions were slightly shifted, as expected. The mean PMU for the repeated condition (491 ms) was slightly larger than the mean PMU for the novel condition (488 ms). Neither a main effect of ISI nor an interaction of the two factors were evident (both *Fs* < 1)^3^.

Taken together, the results of Experiment 1 show that novel stimuli are estimated to be longer than repeated stimuli of the same physical duration. This result was further supported by the RT results which showed that longest RT and thus the greatest uncertainty was observed at slightly shorter comparison durations for novel stimuli than for repeated stimuli. Participants' discrimination sensitivity was neither influenced by stimulus repetition nor by the ISI manipulation.

## 3. Experiment 2

The pseudowords in Experiment 1 did not convey high-level semantic information. Nevertheless, they followed the rules of German orthography and phonology and thus were pronounceable. It is therefore conceivable that the standards were retained in memory by subvocal rehearsal (Baddeley, 2012). This may have influenced the judged duration of repeated as compared to novel comparisons. Former research has shown that “illegal nonwords” like strings of consonants are more difficult to remember (Bowers, 1994) and harder to subvocalize than pseudowords (McCusker et al., 1981). We therefore conducted another experiment in which unpronounceable strings of consonants were used as stimuli.

### 3.1. Method

#### 3.1.1. Participants

A fresh sample of 32 volunteers (19 female, 29 right-handed), aged between 18 and 33 years (*M* = 24.4 years) participated in the experiment. All participants had normal or corrected-to-normal vision, and received course credit or €6. The data of six additional participants was collected but had to be excluded from analyses due to the exclusion criteria already used in Experiment 1. All participants gave informed consent.

#### 3.1.2. Apparatus and stimuli

Experiment 2 was identical to Experiment 1 except for the following changes. A MAC computer controlled stimulus presentation and recorded the participants' responses. The same VGA monitor was used as in Experiment 1. To generate a set of unpronounceable consonant strings, we transformed the set of stimuli from Experiment 1 as follows: Vowels in the pseudowords were replaced by consonants (“Y” was not used as replacement because it is sometimes pronounced like an “I” in German) whereby identical vowels in one pseudoword were replaced by the same consonant and different vowels were replaced by different consonants (e.g., MEILEG was changed to MKPLKG). The assignment from vowel to consonant was randomized for each word (e.g., MEILEG to MKPLKG and SEBSER to SMBSMR). In the end, a set of 104 consonant strings was created for each participant from which 81 were randomly selected to appear in the experiment. The items for the practice block were chosen from the remaining 23 strings of consonants.

#### 3.1.3. Procedure, design, and data analysis

Again, RTs larger than 4000 ms were excluded (146 trials, < 0.8% of all trials).

### 3.2. Results and discussion

**Figure 4:**
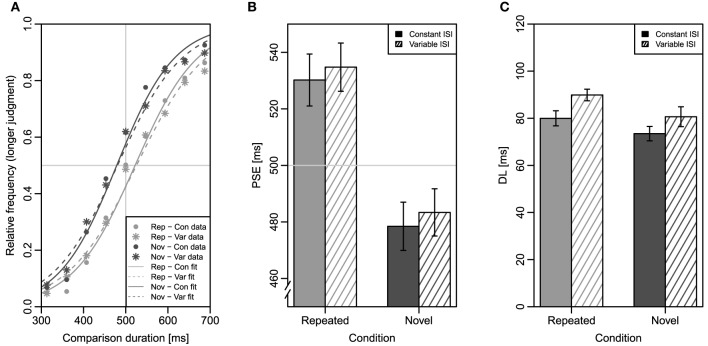
**Results of Experiment 2**. Error bars represent ±1 standard error for within-subjects designs according to Morey (2008). **(A)** Mean relative frequencies of longer judgments as a function of comparison duration in the four conditions (symbols) and fitted logistic functions for all participants' data (lines). Note that this plot is only for illustration; individually fitted logistic functions were the basis of the statistical analyses. The horizontal light gray line indicates the 50% point; the vertical light gray line indicates the standard duration of 500 ms. **(B)** Mean point of subjective equality (PSE) in the four conditions. **(C)** Mean difference limen (DL) in the four conditions. Rep, repeated condition; Nov, novel condition; Con, constant inter-stimulus interval; Var, variable inter-stimulus interval.

Figure 4A shows mean relative frequencies of “longer”-judgments and the fitted logistic functions for all four conditions. Figure 4B depicts mean PSE as a function of the factors repetition and ISI. As in Experiment 1, a significant main effect of repetition was obtained, *F*_(1, 31)_ = 18.90, *MSE* = 4505, *p* < 0.001, $\eta_{p}^{2}=0.38$, indicating again that the mean PSE for repeated comparisons (533 ms) was larger than the mean PSE for novel comparisons (481 ms). As before, the main effect of ISI was not significant, *F* < 1; mean PSE was 504 ms for constant ISIs and 509 ms for variable ISIs. Like in Experiment 1, the ANOVA revealed no interaction of the two factors, *F* < 1^4^.

Mean DL for the four conditions is shown in Figure 4C. The main effect of repetition on DL was significant in this experiment, *F*_(1, 31)_ = 9.70, *MSE* = 204.6, *p* = 0.004, $\eta_{p}^{2}=0.24$, indicating that mean DL was larger for repeated trials (85 ms) than for novel trials (77 ms). There was a marginally significant main effect of ISI, *F*_(1, 31)_ = 3.79, *MSE* = 618.2, *p* = 0.061, $\eta_{p}^{2}=0.11$, reflecting a trend for slightly better discrimination sensitivity when ISIs were constant (77 ms) than when they were variable (85 ms). The interaction of both factors was non-significant, *F* < 1.

**Figure 5:**
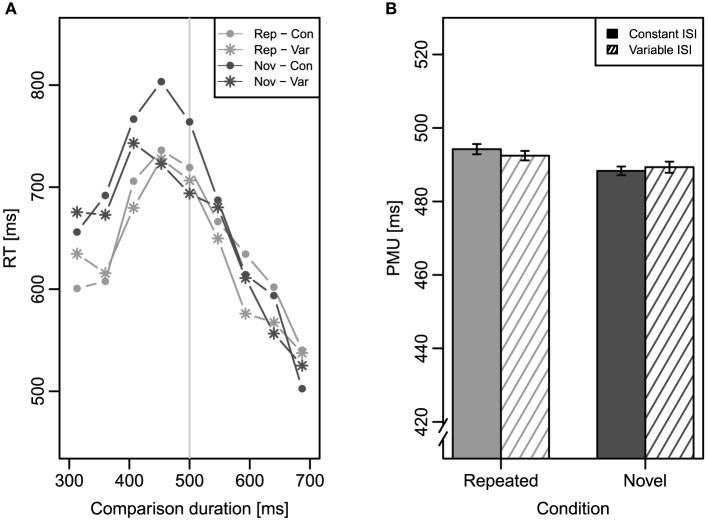
**Results of Experiment 2**. Error bars represent ±1 standard error for within-subjects designs according to Morey (2008). **(A)** Mean response time (RT) as a function of comparison duration, for the four conditions. The vertical light gray line indicates the standard duration of 500 ms. **(B)** Mean point of maximal uncertainty (PMU) for the four conditions. Rep, repeated condition; Nov, novel condition; Con, constant inter-stimulus interval; Var, variable inter-stimulus interval.

Mean RT as a function of repetition condition and comparison duration is illustrated in Figure 5A. Mean RT was again longer for novel (666 ms) than for repeated comparisons (641 ms) resulting in a significant main effect of repetition, *F*_(1, 31)_ = 9.12, *MSE* = 19, 520, *p* = 0.005, $\eta_{p}^{2}=0.23$. As before, no main effect of ISI was observed, *F* < 1, with a mean RT of 663 ms in the constant ISI condition and a mean RT of 666 ms in the variable ISI condition. The main effect of comparison duration was significant, *F*_(8, 248)_ = 23.11, *MSE* = 29, 349, *p* < 0.001, $\eta_{p}^{2}=0.43$, illustrating the typical reversed U-shaped comparison duration-RT function. As in Experiment 1, the factors repetition and comparison duration interacted significantly, *F*_(8, 248)_ = 3.28, *MSE* = 9737, *p* = 0.005, $\eta_{p}^{2}=0.10$. No other effect was significant.

Mean PMU of the four conditions is depicted in Figure 5B. The ANOVA on PMU revealed again a significant main effect of repetition, *F*_(1, 31)_ = 12.82, *MSE* = 51.20, *p* = 0.001, $\eta_{p}^{2}=0.29$, confirming that mean PMU was slightly larger for the repeated condition (493 ms) than for the novel condition (489 ms). Neither a main effect of ISI, *F* < 1, nor an interaction of the two factors was evident, *F*_(1, 31)_ = 2.72, *MSE* = 22.39, *p* = 0.109, $\eta_{p}^{2}=0.08$^5^.

Taken together, the results of Experiment 2 confirm the main finding of Experiment 1, namely that novel stimuli are overestimated in duration as compared to repeated stimuli. The PMU analysis further supported this finding by showing that the comparison durations causing the longest RTs were smaller for novel than for repeated strings of consonants, meaning that the largest uncertainty was observed at shorter comparison durations for novel than for repeated stimuli. In contrast to Experiment 1, discrimination sensitivity benefitted from novel comparisons and marginally from a fixed temporal structure (ISI).

## 4. General discussion

Two experiments were conducted in order to examine the influence of repetition on duration estimation with nonword stimuli. To this end, two nonwords were presented consecutively on each trial and the participants' task was to judge whether the second nonword (comparison) was shorter or longer than the first nonword (standard). Crucially, the comparison could either be a repetition of the standard (repeated comparison) or a different nonword (novel comparison). In Experiment 1, semantically low-associative pseudowords served as stimuli. In Experiment 2, unpronounceable strings of consonants were used as stimuli to test whether the results for pseudowords would transfer to stimulus material for which subvocalizing was not possible.

The results of the two experiments showed that the duration of the comparison was judged to be shorter for repeated than for novel stimuli, thereby replicating and extending the findings of Matthews (2011) who used a similar paradigm with more complex stimulus material (namely photographs of natural or social scenes, objects, and buildings). In the study by Matthews (2011), the effect might have been based on the repetition (for repeated comparisons) or change (for novel comparisons) of multiple low-level and high-level features. In a more recent study (Matthews, 2015), the effect was replicated for abstract stimuli, but these stimuli still contained a variety of different low-level features (like different colors and contrasts) and were constructed from various line drawings. The present study shows that the repetition or change of a simple, meaningless letter string was sufficient to generate the effect. The nonwords we used in the present experiments were well-controlled in low-level features (constant length, white font on black background), composed of familiar features (i.e., letters), and contained no obvious semantic information. Nevertheless, repeating these simple stimuli resulted in shorter judged durations as compared to presenting a different nonword as comparison. Thus, the difference in duration judgments for repeated and novel stimuli seems to be independent of the information complexity the stimuli contain.

Furthermore, immediate stimulus repetition influenced duration judgments irrespective of whether the nonwords were pronounceable (pseudowords) or unpronounceable (strings of consonants). The very similar results of Experiments 1 and 2 illustrate that the possibility to subvocalize the pseudowords and therefore to potentially experience increased processing fluency for repeated pseudowords (Johnston et al., 1985) is not crucial for duration judgment.

These consistent duration judgment results were further supported by RT analyses. It is assumed that participants generally respond faster the more certain they are about a discrimination judgment (Birren and Botwinick, 1955; Sternberg, 1969). Accordingly, comparison durations which are subjectively similar to the standard duration should result in the longest RTs. To determine whether these points of maximal uncertainty were shifted against each other depending on whether comparisons were repeated or novel, we used the waveform moment analysis (Cacioppo and Dorfman, 1987) to determine the means of the comparison duration-RT functions. Indeed, the calculated PMUs were significantly smaller for novel than for repeated comparisons. Thus, participants experienced shorter durations as equivalent to the standard duration when the comparison was novel than when the comparison was repeated. Hence, the PMU analyses complemented the duration judgment results and corroborated that repeated comparisons were perceived as being shorter than novel comparisons.

Moreover, RTs were generally longer for novel than for repeated comparisons. We assume that this main effect does not necessarily reflect lower decision certainty for novel than for repeated trials because discrimination sensitivity (DL) was either equal in the two conditions (Experiment 1) or even superior for novel comparisons (Experiment 2). This finding might rather reflect general processing advantages for repeated stimuli (Pashler and Baylis, 1991; Bentin and McCarthy, 1994) which could be independent from the quality of temporal discrimination. It should be noted, however, that RT advantages for repeated stimuli are usually reported for tasks in which participants have to judge the identity of a stimulus, whereas in our case the identity of the stimulus is actually irrelevant for the response. Therefore, it is not entirely clear, whether the same mechanism are at work both in stimulus discrimination and duration discrimination when stimuli are repeated.

Additionally we were interested in whether a predictable time course within each trial (i.e., a constant ISI between the standard and the comparison) would improve the participants' temporal discrimination sensitivity. This was suggested by Tse et al. (2004) who argued that a constant ISI might induce rhythm perception which could alter duration judgments. To investigate this issue, a constant ISI was used in one half of both experiments while ISIs varied slightly in the other half of both experiments. Only in Experiment 2 did participants show a tendency to discriminate durations better when a predictable time course was used. By and large, however, manipulation of ISI had little if any effect in the present experiments. It could be speculated that a rhythm might only be induced by stream-based paradigms in which a series of standards is presented prior to the comparison. Alternatively, one could argue that the variable ISIs in the present experiments only varied slightly (247–380 ms) in duration; a range which might not have disrupted the anticipation of the comparison onset enough. It is also conceivable that rhythm effects are generally more pronounced for empty time intervals which are defined by two markers at the beginning and the end of a stimulus than for filled intervals which were used in the present study.

Generally, it is well-known in experimental psychology that the repetition of stimuli influences their cognitive processing. The repetition of stimuli has often been understood as a special case of priming (Henson, 2003; Schacter et al., 2007) whereby repetition priming has shown to decrease reaction times and increase discrimination performance for repeated stimuli (Bentin and McCarthy, 1994). The neural mechanism of “repetition suppression,” which describes a reduced neural response to repeated stimulus presentation, has been argued to be responsible for at least some of the behavioral effects of stimulus repetition (Wig et al., 2005). Since the size of the neural response has also been suggested to form the basis of duration perception (Pariyadath and Eagleman, 2012), the present results might be linked to repetition suppression.

Interestingly, there is evidence that this mechanism is not necessarily limited to one-on-one repetitions following an all-or-nothing principle, but that gradual differences between standards and comparisons also shape duration judgments. Specifically, the size of the oddball effect increases the more the comparisons deviate from the standards (Schindel et al., 2011; Pariyadath and Eagleman, 2012). Furthermore, it has been shown that not only repetition but also expectation can influence duration judgments. For example, Pariyadath and Eagleman (2007) reported that the duration of a number embedded in a predictable sequence (e.g., 1 2 3 4) was judged as similarly long as a number embedded in a sequence of repeated numbers (e.g., 1 1 1 1) but as shorter than a number embedded in an unpredictable sequence (e.g., 1 3 5 2). Recent results by Matthews (2015) suggest that the effects of repetition and expectation on duration judgment interact in a complex manner. Surprisingly, Matthews reported smaller repetition effects for frequent than for infrequent repetitions when the likelihood of stimulus repetition was manipulated. Future research needs to further disentangle the effects of repetition on the one hand and expectation on the other hand.

The present study replicates and extends previous findings concerning the effect of immediate stimulus repetition on duration perception. Furthermore, the results clearly suggest that changes of simple, meaningless stimuli with similar low-level features are sufficient to induce a shorter perceived duration of repetitions, and that the temporal structure within the reminder task has no pronounced effect on duration judgments.

### Conflict of interest statement

The authors declare that the research was conducted in the absence of any commercial or financial relationships that could be construed as a potential conflict of interest.

## Footnotes

^1^Consider *m*_*i*_ being the mean RT at comparison duration *d*_*i*_, *i* = 1, …, 9. First, these means are scaled as $m_{i}^{*}=\frac{m_{i}}{\sum_{i=1}^{9}m_{i}},\;i=1,\ldots ,n$, *i* = 1, …, *n*. Second, these scaled values are used to compute $M=\sum_{i=1}^{9}d_{i}\cdot m_{i}^{*}$, that is, the location of the observed comparison duration-RT function.

^2^As mentioned before, the multiple presentation of the pseudowords throughout the experiment should not have influenced the effect of repetition condition on PSE because this effect is assumed to be short-lived. Nevertheless, we performed an additional repeated measures ANOVA with the factors repetition and experimental half (first half vs. second half) on PSE to check whether the repetition effect changed over time and hence with increasing number of stimulus presentations. The main effect of condition was identical to the one in the main analysis, *F*_(1, 31)_ = 23.60, *MSE* = 1975, *p* < 0.001, $\eta_{p}^{2}=0.43$. Although there was a significant main effect of experimental half on PSE, *F*_(1, 31)_ = 8.10, *MSE* = 593, *p* = 0.008, $\eta_{p}^{2}=0.21$ (first half: 501 ms, second half: 489 ms), the repetition effect was of similar size in the first (36 ms) and in the second half of the experiment (41 ms), *F* < 1.

^3^Additionally, we used an alternative method to estimate PMU. We fitted second degree polynomials to the comparison duration-RT functions of each condition and for each participant and determined their maxima. The comparison duration at which the maximum was located served as *PMU*_*poly*_. Qualitatively, we observed the same pattern of results for *PMU*_*poly*_ as for PMU.

^4^Again an additional repeated measures ANOVA with the factors repetition and experimental half was conducted on PSE to check whether the repetition effect changed over the experimental course. The main effect of condition was identical to the one in the main analysis, *F*_(1, 31)_ = 18.90, *MSE* = 4505, *p* < 0.001, $\eta_{p}^{2}=0.38$. A significant main effect of experimental half, *F*_(1, 31)_ = 17.21, *MSE* = 1530, *p* < 0.001, $\eta_{p}^{2}=0.36$, demonstrated that PSE decreased from the first (521 ms) to the second half (481 ms) of the experiment. But importantly, the repetition effect was of almost equal size in the first (53 ms) and in the second half (51 ms) of the experiment, *F* < 1.

^5^As in Experiment 1, the additional analysis of *PMU*_*poly*_ yielded qualitatively the same pattern of results.
